# Emerging Biomarkers in Thyroid Practice and Research

**DOI:** 10.3390/cancers14010204

**Published:** 2021-12-31

**Authors:** Shipra Agarwal, Andrey Bychkov, Chan-Kwon Jung

**Affiliations:** 1 Department of Pathology, All India Institute of Medical Sciences, New Delhi 110608, India; drshipra0902@gmail.com; 2 Department of Pathology, Kameda Medical Center, Kamogawa 296-8602, Chiba, Japan; 3 Department of Hospital Pathology, College of Medicine, The Catholic University of Korea, Seoul 06591, Korea; 4 Cancer Research Institute, College of Medicine, The Catholic University of Korea, Seoul 06591, Korea

**Keywords:** thyroid cancer, molecular, liquid biopsy, targeted therapy, immunohistochemistry, tumor microenvironment, diagnosis, prognosis, predictive biomarkers

## Abstract

**Simple Summary:**

Tumor biomarkers are molecules at genetic or protein level, or certain evaluable characteristics. These help in perfecting patient management. Over the past decade, advanced and more sensitive techniques have led to the identification of many new biomarkers in the field of oncology. A knowledge of the recent developments is essential for their application to clinical practice, and furthering research. This review provides a comprehensive account of such various markers identified in thyroid carcinoma, the most common endocrine malignancy. While some of these have been brought into use in routine patient management, others are novel and need more research before clinical application.

**Abstract:**

Thyroid cancer is the most common endocrine malignancy. Recent developments in molecular biological techniques have led to a better understanding of the pathogenesis and clinical behavior of thyroid neoplasms. This has culminated in the updating of thyroid tumor classification, including the re-categorization of existing and introduction of new entities. In this review, we discuss various molecular biomarkers possessing diagnostic, prognostic, predictive and therapeutic roles in thyroid cancer. A comprehensive account of epigenetic dysregulation, including DNA methylation, the function of various microRNAs and long non-coding RNAs, germline mutations determining familial occurrence of medullary and non-medullary thyroid carcinoma, and single nucleotide polymorphisms predisposed to thyroid tumorigenesis has been provided. In addition to novel immunohistochemical markers, including those for neuroendocrine differentiation, and next-generation immunohistochemistry (BRAF V600E, RAS, TRK, and ALK), the relevance of well-established markers, such as Ki-67, in current clinical practice has also been discussed. A tumor microenvironment (PD-L1, CD markers) and its influence in predicting responses to immunotherapy in thyroid cancer and the expanding arena of techniques, including liquid biopsy based on circulating nucleic acids and plasma-derived exosomes as a non-invasive technique for patient management, are also summarized.

## 1. Introduction

Thyroid nodules are common in the general population, with a higher prevalence noted in women and the older age group. While palpable thyroid nodules account for less than 10% of the adult population, high-resolution ultrasound can detect subcentimeter nodules and clinically silent nodules, increasing the prevalence to about 70% [1,2]. Although the vast majority of thyroid nodules are non-neoplastic, 5–25% of thyroid nodules are true neoplasms [3,4].

The spectrum of thyroid neoplasms ranges from benign follicular adenoma (FA) to low-risk neoplasms with borderline or uncertain behavior to malignancies [5]. Low-risk neoplasms include non-invasive follicular thyroid neoplasms with papillary-like nuclear features (NIFTP), thyroid tumors of uncertain malignant potential, and hyalinizing trabecular tumors. Of the malignant thyroid tumors of a follicular cell origin, differentiated thyroid carcinomas (DTCs) include papillary thyroid carcinomas (PTCs), comprising 80–90% of cases; follicular thyroid carcinomas (FTCs); oncocytic (Hürthle cell) carcinomas (OCAs); and poorly differentiated thyroid carcinomas (PDTCs). Anaplastic thyroid carcinoma (ATC) is an undifferentiated malignancy of follicular thyroid cell origin, showing one of the most aggressive clinical behaviors among human cancers. Medullary thyroid carcinoma (MTC) originates from parafollicular C cells producing calcitonin.

As thyroid cancers encompass a broad gamut of tumors differing in their molecular, histologic features and clinical behavior, there is a need for identifying robust biomarkers for accurate diagnosis and management. Such markers will also be helpful in improving the preoperative categorization of thyroid nodules, 15–30% of which remain diagnostically challenging to fine needle aspiration (FNA) specimens [6].

Cancer biomarkers can be genetic materials, proteins, chemical modifications and characteristics that can be measured through clinical, pathological, radiological and other features. Recent and continuing developments, including the use of high-throughput technologies, have led to the identification of many new biomarkers in the field of thyroid cancer. These not only aid in accurate and, in some cases, early diagnosis, but also provide useful information in guiding clinical decision-making in patients with thyroid cancer. This review provides a comprehensive overview of the recent advances in genetic and epigenetic alterations and protein expression as biomarkers for thyroid neoplasms.

## 2. Molecular Landscape of Follicular Cell-Derived Thyroid Cancer

**Figure 1:**
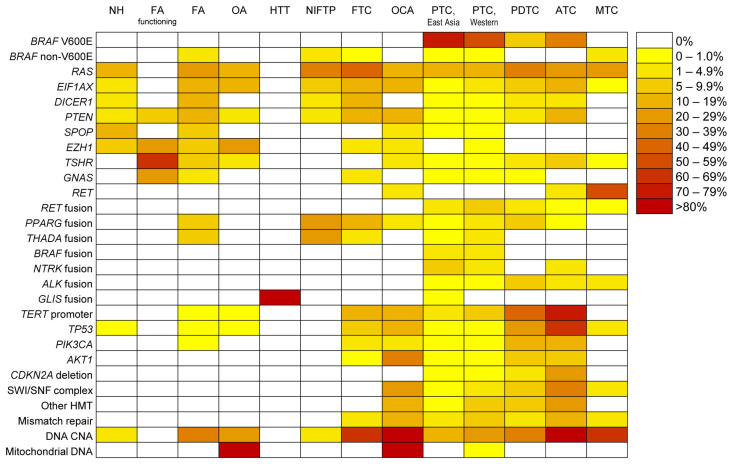
Frequency and pattern of genetic alterations across thyroid tumors. The heatmap depicts the frequency of non-synonymous mutations, deletions and fusions in selected genes, and copy number alterations (CNA). NH, nodular hyperplasia; FA, follicular adenoma; OA, oncocytic adenoma; NIFTP, non-invasive follicular thyroid neoplasm with papillary-like nuclear features; HTT, hyalinizing trabecular tumor; FTC, follicular thyroid carcinoma; OCA, oncocytic carcinoma; PTC, papillary thyroid carcinoma; OCA, oncocytic carcinoma; PDTC, poorly differentiated thyroid carcinoma; ATC, anaplastic thyroid carcinoma; MTC, medullary thyroid carcinoma; and HMT, histone methyltransferase. References for information used in this figure can be found in Table S1.

The Cancer Genome Atlas (TCGA) study identified oncogenic driver alterations for 96.5% of studied PTC cases [7]. Most genetic alterations occurred in a limited number of genes and led to a low mutation burden, compared to other carcinomas. These alterations frequently occur in a mutually exclusive manner. The most frequent oncogenic alteration in PTC was *BRAF* V600E (60%), followed by *NRAS* mutations (8.5%) and *RET* gene fusions (6.3%). Other genetic alterations found in less than 5% of cases include mutations of *HRAS*, *KRAS*, *EIF1AX*, *PPM1D*, *CHEK2*, *ARID1B*, *PTEN* and, *TP53*, and gene fusions of *BRAF*, *PPARG, NTRK1, NTRK3, ALK*, and *THADA* (Figure 1).

In the TCGA cohort, PTCs were classified into 2 molecular subtypes based on a 71-gene expression signature: *BRAF* V600E-like and *RAS*-like PTCs. The molecular subtype of PTCs can be further subclassified into three groups (*BRAF*-like, *RAS*-like, and non-*BRAF*/non-*RAS*) [8]. The non-*BRAF*/non-*RAS* subtype was associated with less aggressive thyroid cancer, with a follicular growth pattern and mutations of *EIF1A*, *PTEN*, *DICER1*, *EZH1*, *SOS1*, *IDH1*, and *SPOP*, *PPARG* fusion, and *THADA* fusion [8]. The immune-related metagene signature representing expression levels of immune-related genes categorized the canonical *BRAF* V600E-like and *RAS*-like PTCs into four groups (*BRAF*-like immunodeficient, *BRAF*-like immunoreactive, *RAS*-like immunodeficient, and *RAS*-like immunoreactive) [9]. In patients with PTC, the *BRAF*-like immunoreactive subtype was associated with tall cell variants and worst prognosis, while the *RAS*-like immunodeficient subtype was enriched in follicular variant tumors [9].

The most frequent mutation detected in FA and FTC occurs in *RAS* genes (*NRAS*, *HRAS*, and *KRAS*), followed by mutations of *DICER1*, *EZH1*, *EIF1AX*, *PTEN*, *IDH1*, and *SPOP*, and *PPARG* gene fusion (Figure 1) [8]. There is no significant difference in the mutation profiles between FA and FTC [8,10]. Nodular hyperplasia (NH) morphologically mimics FA, and the two can be difficult to differentiate. However, a growing number of reports have documented *RAS* mutations in NH [11,12,13]. A recent publication implicated additional alterations in the expressions of genes involved in cell cycle, apoptosis, and PI3K pathway, and stromal factors to lead to a stepwise progression of NH to malignancy [12].

**Figure 2:**
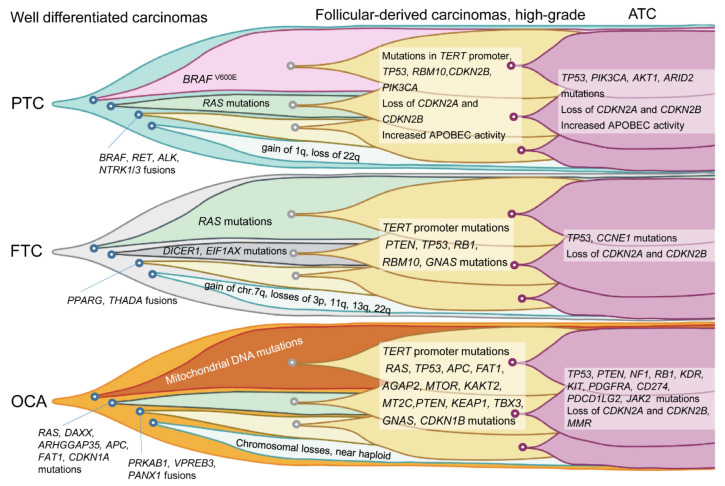
Genetic evolution of differentiated thyroid cancers. PTC, papillary thyroid carcinoma; FTC, follicular thyroid carcinoma; OCA, oncocytic carcinoma; and ATC, anaplastic thyroid carcinoma.

Compared to DTC, PDTC and ATC have a higher tumor mutation burden and a higher frequency of mutations involving *TERT* promoter; tumor-suppressor genes, including *TP53*; PI3K/AKT/mTOR pathway genes; cell-cycle genes, including *CDKN2A*, *CDKN2B*, and *CCNE1*; and genes associated with SWI/SNF nucleosome remodeling and histone modification (Figure 1) [14,15]. The progression of DTC to a more aggressive form, PDTC, and ATC is associated with the accumulation of mutations in various genes (Figure 2) [15].

### 2.1. Recently Discovered Molecular Alterations in Thyroid Cancer

#### 2.1.1. Recurrent Promoter Mutations in Thyroid Cancer

Whole-genome sequencing of thyroid tumors has identified recurrent driver mutations in non-coding regulatory regions, such as gene promoters. Two hotspot mutations in the *TERT* promoter region at chr. 5:1,295,228 (C228T mutation) and 1,295,250 (C250T mutation) were found to be associated with tumor progression and poor clinical outcomes of thyroid cancer. *TERT* promoter mutations occur in 10–20% of DTC, 40% of PDTC, and 65% of ATC [14,15,16].

Two hotspot mutations in the *PLEKHS1* promoter region at chr. 10: 115,511,590 (G590A mutation) and chr. 10: 115,511,593 (C593T), occurred in 10% of DTC with distant metastasis and were proposed as genetic markers of aggressive DTC [16].

#### 2.1.2. ALK

*Anaplastic lymphoma kinase (ALK)* gene translocations have been implicated as pathogenic events in a proportion of PTC (0–9%), PDTC (0–4%), and ATC (0–4%) [7,17,18,19], with some reports indicating a greater prevalence in dedifferentiated thyroid cancers [17]. Common reported fusion partners include *EML4* and *STRN*, others being *TFG*, *GTF2IRD1*, and *CCDC149* [19].

*ALK* translocations appear to be more frequent post-radiation [20], in female PTC patients [19,21], have been variably reported in the pediatric age group [22], and occur independently of other known driver molecular alterations in thyroid cancers [17,21].

In addition to translocations, *ALK* mutations have also been reported in 11–20% of ATC [23,24], and can be associated with *NF1* and/or *TP53* mutations [23].

Another tumor linked with *ALK* gene fusions and rarely reported in the thyroid gland is the inflammatory myofibroblastic tumor [25].

Both translocations and mutations result in an enhanced activity of ALK kinase, which can be detected by ALK immunohistochemistry (IHC) [20,21], and is amenable to inhibition by ALK inhibitors, such as crizotinib [17].

#### 2.1.3. NTRK

Various fusion proteins formed, due to translocations involving any of the *neurotrophic-tropomyosin receptor kinase* (*NTRK*) genes (*NTRK1*, *NTRK2*, and *NTRK3*) with other genes, is a known oncogenic event in many tumors. These are located, respectively, on chromosomes 1q23.1, 9q21.33, and 15q25.3, and encode the transmembrane receptor proteins TrkA, TrkB, and TrkC. *NTRK1* and *NTRK3* are the genes usually involved. The reported oncogenic fusion partners in thyroid tumors include *PPL*, *EML4*, *ETV6*, *IRF2BP2*, *TPR*, *TPM3*, *TFG*, *TRIM33*, *RBPMS*, *SQSTM1*, and *ERC1*. *NTRK* rearrangements lead to the constitutive activation of the intracellular tyrosine kinase domain of the receptor. This causes cellular proliferation and tumorigenesis following uncontrolled downstream pathway activation. TrkA activates the MAPK pathway, TrkB the Ras-ERK, PIK3, and PLC-gamma pathways, and TrkC the PI3/AKT pathway [26].

While more frequent in other solid tumors, in the thyroid, these rearrangements are observed in about 2.3% of the sporadic adult-onset carcinomas [27,28,29], with higher frequencies reported in post-radiation exposure (3–15%) [30,31] and in the pediatric age group (2–26%) [22,32].

*NTRK* rearrangements have been associated with PTC and rarely with primary thyroid secretory carcinomas [33]. These PTCs can show a multinodular infiltrative growth pattern, or a predominant follicular pattern variably admixed with papillae, or the solid growth pattern [33,34]. Variably reported features include glomeruloid structures, focal cytoplasmic clearing, and reverse nuclear polarity [27,34]. *NTRK*-rearranged thyroid carcinomas often show extensive lymphovascular invasion, extrathyroidal extension, and lymph node metastases. Although they can develop distant metastases, mortality rates are low [33,34].

TRK inhibitors entrectinib and larotrectinib, have been approved by the United States Food and Drug administration (FDA) for use in patients with solid tumors harboring *NTRK* fusions [35].

*NTRK* rearrangements can be detected by various molecular techniques, including fluorescence in situ hybridization (FISH), reverse-transcriptase PCR (RT-PCR), next generation sequencing (NGS), and IHC, all with their respective advantages and shortfalls [26].

#### 2.1.4. DICER1

DICER1 is a highly conserved RNaseIII endoribonuclease, involved in the synthesis of microRNAs (miRNAs) and short interfering RNAs (siRNAs); therefore, it has a critical role in post-transcriptional gene silencing [36]. Loss of function mutations of the *DICER1* gene have an oncogenic effect, with a particular predisposition to childhood-onset tumors [37]. Germline mutation leads to an autosomal-dominant disorder, the DICER1 syndrome. A second somatic mutation in these patients is linked with a wide spectrum of benign and malignant lesions involving various organ systems, the most frequent being pleuropulmonary blastoma, multinodular goiter (MNG), cystic nephroma, and ovarian sex cord-stromal tumors [38].

Most patients are female carriers in the age group of 10 to 30 years [38]. In a recent study, 75% of women and 17% of men with DICER1 syndrome developed MNG or underwent thyroidectomy by 40 years of age [39]. It has been proposed that germline *DICER1* mutations are predisposed to thyroid follicular cell hyperplasia and the subsequent acquisition of one or more somatic RNase IIIb mutations results in the development of multiple thyroid nodules and MNG [40]. DICER1 syndrome should be suspected if MNG occurs in childhood, in a patient with an ovarian Sertoli –Leydig cell tumor or when familial [41,42].

It also increases the risk of thyroid cancer by 16- to 24-fold [39]. It has been suggested that DTC develops in *DICER1* variant carriers, following a stepwise acquisition of mutations involving somatic mutations of *DICER1* and additional molecular events, distinct from the pathways observed in PTC and FTC [38,43]. A recent study found that about 50% of pediatric FTCs had *DICER1* mutations [44]. The mutation rate in pediatric PTC is 10%, compared to approximately 0.5% in adults [45]. *DICER1* alterations are, hence, driver mutations in at least a proportion of adolescent-onset benign and malignant thyroid tumors [37,45]. Most of the thyroid cancers reported, to date, have been low-risk malignancies, particularly the follicular variant of PTC and FTC [39]. An association has also been found with macrofollicular patterned neoplasms [46]. Some recent publications, in which the NGS platform was used, documented somatic DICER1 mutations in OCA, and the aggressive subtypes of thyroid cancers PDTC and ATC [47,48].

For surveillance purposes, major and minor indications for considering germline *DICER1* genetic testing have been recently proposed. MNG or DTC, in two or more first-degree relatives, or in an index case with a family history consistent with DICER1 syndrome, is a major indication, as is a case of childhood-onset MNG or DTC [49].

An aggressive tumor linked with *DICER1* mutation, but not with DICER1 syndrome, is malignant teratoma of the thyroid gland, and the term “thyroblastoma” has been proposed for this entity [50,51].

#### 2.1.5. PTEN

*Phosphatase and tensin homolog deleted on chromosome ten* (*PTEN*) is a tumor suppressor gene located at chromosome 10q23.3. *PTEN* hamartoma tumor syndrome is an autosomal dominant tumor susceptibility syndrome, resulting from heterozygous germline pathogenic variants of *PTEN*. It encompasses multiple disorders, including Cowden syndrome, Bannayan–Riley–Ruvalcaba syndrome, *PTEN*-related Proteus syndrome, and *PTEN*-related Proteus-like syndrome. Clinical features include neurological disorders, multifocal hamartomas, and lifetime predilection, to develop malignancies in various organ systems (Cowden syndrome) [49,52,53].

Patients with *PTEN* hamartoma tumor syndrome, develop both benign and malignant thyroid lesions at a higher frequency than in the general population. Multiple pathologies can be present in the same thyroidectomy specimen, such as multiple FA [54], and MNG in a background of thyroiditis [55,56]. While FTC is one of the major criteria recommended by NCCN for the diagnosis of PTEN hamartoma tumor syndrome, PTC or its follicular variant and other thyroid structural lesions, such as adenoma, and MNG are included among the minor criteria [52].

Thyroid tumors can also harbor sporadic *PTEN* inactivation, secondary to deletion, mutation, or promoter methylation [57,58,59,60]. In a recent study, of the three evaluated PTC subtypes/variants, classical, follicular, and tall cell, the loss of PTEN protein immunoexpression was reported to be more frequent in the follicular variant [58].

#### 2.1.6. GLIS

The GLISimilar (GLIS) proteins (1–3) are a family of the GLI-similar zinc finger transcription factors, which act as transcriptional activators and repressors. While the physiological role of GLIS1 in thyroid cells is not well established, GLIS3 regulates thyroid gland development [61] and thyroid hormone synthesis [62,63]. Interchromosomal rearrangements involving *GLIS3*, located on chromosome 9p24.2 and less commonly *GLIS1* on chromosome 1p32.3, are pathognomonic of the hyalinizing trabecular tumor (HTT) of the thyroid [64,65]. There is the juxtaposition of exon 3 of *GLIS3* or exon 2 of *GLIS1* downstream of exon 2 of *PAX8* on chromosome 2q14.1, placing their zinc-finger containing DNA-binding domains under the regulation of *PAX8* promoter [65], and resulting in the constitutive activation of GLIS. Further downstream pathways that lead to HTT tumorigenesis have not been completely established, although there is limited evidence implicating the activation of the Sonic Hedgehog pathway [66], and the upregulation of extracellular matrix-related genes [65].

Recently, GLIS3 protein overexpression has been demonstrated to be detectable by IHC, appearing as combined nuclear and cytoplasmic positivity [65].

#### 2.1.7. EIF1AX

Eukaryotic initiation factor 1A, X-linked (*EIF1AX*), is essential for the initiation of the translation process. Its role as a pathogenetic event in thyroid tumor genesis was first demonstrated by TCGA study, whereby *EIF1AX* mutations were found in 1.5% of PTC lacking any other known driver mutations [7]. *EIF1AX* alterations have been reported in thyroid lesions, other than PTC, including FTC, PDTC, ATC, and FA, and in the hyperplastic thyroid nodule (limited evidence in benign lesions) [14,67,68]. The reported prevalence for *EIF1AX* mutations has ranged from 0.3% [69] to 2.3% [68] in PTC, 0–5.1% in FTC [68,70], 11–22% in PDTC [14,71], and 9–14% in ATC [14,67].

Interestingly, *EIF1AX* mutations have been found to co-occur with *RAS* mutations in PDTC and ATC [14,67,71,72], but not with *BRAF* or *TERT* promoter mutations [14,67]. Functional studies have shown that the *EIF1AX-A113* splice site mutation, which is the most frequent, via the induction of activating transcription factor 4 (ATF4), inhibits phosphorylation of EIF2α, increasing protein synthesis. ATF4, along with c-MYC, the latter stabilized by RAS, sensitizes mTOR to the amino acid supply, thus increasing therapeutic susceptibility to MEK, BRD4, and mTOR kinase inhibitors [72].

### 2.2. Epigenetics

Major epigenetic mechanisms that deregulate gene expression and can contribute to carcinogenesis, include DNA methylation, histone modification, and non-coding RNA species. The latter are represented by microRNAs (miRNA) and long non-coding RNAs.

#### 2.2.1. DNA Methylation

The aberrant DNA methylation of promoters and enhancers affects gene expression. Hypermethylation can lead to the silencing of tumor suppressor genes, leading to carcinogenesis; common examples in thyroid cancer include Ras association domain family 1; isoform A (*RASSF1A*); cyclin-dependent kinase inhibitor 2A (*CDKN2A* or *P16INK4A*); death-associated protein kinase1 (*DAPK*); tissue inhibitor of metalloproteinase-3 (*TIMP3*); *SLC5A8*; *SLC5A5*; thyroid stimulating hormone receptor (*TSHR*); *PTEN*; retinoic acid receptor β2 (*RARβ2*); RAP1 GTPase activating protein (RAP1GAP); and fibroblast growth factor receptor (FGFR) 2 [73,74,75,76]. Targeted methylation analysis showed that PTC is more likely to exhibit hypomethylation than hypermethylation, compared with a normal thyroid; this is contrasted to FTC, which displays more hypermethylations than hypomethylations [76,77]. However, an evaluation of the global methylation status found that hypermethylation was detected in all well-differentiated thyroid neoplasms (FA, FTC, and PTC), compared to the adjacent non-neoplastic thyroid tissue [78].

Hypomethylation of cytosine-guanine dinucleotides (CpG) islands, located in the promoter of *MMP7* and in the gene bodies of *MICAL2* and *DIAPH1*, have been suggested to be helpful in the differential diagnosis of non-malignant thyroid tumors from DTC. In PTC, the hypomethylation of these markers has also been associated with *BRAF* V600E mutation, lymph node metastasis, extrathyroidal extension, distant metastasis, and recurrent/persistent disease [77].

Protein expression of thyroid transcription factor-1 (TTF-1), a commonly used immunohistochemical marker for thyroid cell differentiation, is often absent in ATC, unlike DTC. The loss in expression has been explained by the DNA methylation and histone H3 modification of the gene encoding TTF-1, *NK2 homeobox 1* (*NKX2-1*) [73,74].

Demethylating drugs are currently under evaluation, for their utility in the management of thyroid cancer patients who are refractory to radioactive iodine (RAI). They inhibit DNA methyl transferases, leading to the reactivation of silenced genes. The specific mechanisms of action in thyroid cancer include the restoration of the expression of sodium/iodide symporter, and thus of RAI uptake [75].

The role of histone modifications in thyroid carcinogenesis has also been evaluated, but to a limited extent. Their importance is highlighted by the utility of histone deacetylase (HDAC) inhibitors in improving the uptake of RAI in ATC. Histone modification acts by multiple mechanisms in thyroid carcinogenesis; for example, the repression of expression of *paired-box gene 8* (*PAX8*), a thyroid-specific transcription factor, induction of loss of cell cycle regulation, and dedifferentiation [74].

Another epigenetic event implicated in aggressive thyroid carcinomas is chromatin remodeling via mutations in the Switch/Sucrose Non-Fermentable (SWI/SNF) complex [74].

#### 2.2.2. MicroRNA

MicroRNAs (miRNA) are small non-coding RNAs involved in the post-transcriptional regulation of gene expression. In pathological conditions, in addition to playing a role in pathogenesis, they act as biomarkers for diagnostics, prognostication, and for the follow-up of patients with malignancies, including thyroid cancer. Circulating levels of miRNAs, in addition, provide a non-invasive tool [79].

The miRNAs that have been shown to be consistently upregulated in PTC tissues, when compared with non-neoplastic thyroid, include miR-21, miR-127, miR-136, miR-146b, miR-221, miR-222, and miR-181b [79,80,81,82,83]. MicroRNAs miR-221 and miR-222 negatively regulate p27 protein, a cyclin-dependent kinase inhibitor and a cell-cycle regulator [84]. Similarly, miR-146b and miR-181b target the tumor suppressor genes, retinoic acid receptor beta, and CYLD, respectively [85,86]. Importantly, in the PTC series, the overexpression of miR-221, miR-222, and miR-146b has been associated with adverse clinicopathological features, such as extrathyroidal invasion, lymph node and distant metastasis, advanced disease stage, recurrence, and *BRAF* V600E mutation [82]. The upregulation of miR-136, miR-21, and miR-127 was associated with distant metastases and recurrent/persistent disease in DTC [83]. Moreover, there is evidence suggesting the downregulation of expression of certain miRNAs in PTC. These include miR-145, miR-451, miR-613, and miR-137 [82].

Limited data is available regarding the miRNA expression profiling of FTC. Similar to PTC, miR-146b and miR-221 have been found to be upregulated in FTC [87]. A study analyzing miRNA expression in FTC and OCA, reported the upregulation of miR-182, miR-183, miR-221, miR-222, and miR-125a-3p, and the downregulation of miR-542-5p, miR-574-3p, miR-455, and miR-199a in both, when compared to normal thyroid tissue. The authors also documented miR-885-5p to be upregulated in OCA, but not in FTC [88]. The miRNA miR-199a-5p, suppresses the function of the connective tissue growth factor in FTC, thus acting as a tumor suppressor [89]. Sparse data exists on the prognostic role of miRNAs in FTC. Metastatic FTCs and widely invasive FTCs have been shown to be more likely to have higher levels of miR-221-3p, miR-222-3p, miR222-5p, miR-10b, and miR-92a. Furthermore, limited data suggests miR-10b to be a potential tool for predicting the metastatic potential of minimally invasive FTC [90].

As seen in DTC, ATC too shows the upregulated expression of miR-146b, miR-221, and miR-222. In contrast, the downregulation of miRNAs of miR-200 (miR-200a, miR-200b, and miR-200c) and miR-30 (miR-30a, miR-30b, miR-30c, miR-30d, and miR-30e) families is specific for ATC. Both these miRNAs regulate the epithelial–mesenchymal transition [82,91].

MTC also shows an aberrant expression of multiple miRNAs. Of note, the increased expression of miR-21, miR-183, and miR-375 has been associated with poor clinical outcomes, in terms of lymph node and distant metastasis, residual disease, advanced tumor stage, and mortality [92,93,94].

The thyroid miRNA classifier (ThyraMIR) is a commercial molecular diagnostic test for indeterminate thyroid cytology results to rule in all types of thyroid cancers, based upon the relative expression of 10 miRNAs: miR-222-3p, miR-146b-5p, miR-375, miR-29b-1-5p, miR-31-5p, miR-138-1-3p, miR-139-5p, miR-155, miR-204-5p, and miR-551b-3p [95].

#### 2.2.3. lncRNA

Long non-coding RNAs (lncRNAs) are typically smaller than mRNA, but greater than 200 nucleotides in length and do not code for proteins. Long non-coding RNAs epigenetically regulate the expression of genes involved in cell cycle, cell differentiation, proliferation, apoptosis, migration, and invasion. Consequent to the alteration of their expression, they can act as oncogenes or tumor suppressor genes. Similar to miRNAs, lncRNAs are potential diagnostic and prognostic cancer markers, as well as therapeutic targets. They can be measured both in tissues and in blood.

Recent studies have established that deregulation of lncRNAs contributes to thyroid cancer development and behavior. While some of them act as tumor suppressors (including, GAS8-AS1, LINC00271, LINC003121, MEG3, NAMA, NONHSAG007951, NONHSAG018271, NONHSAT037832, and PTCSC1/2/3), others act as oncogenes (namely, ANRIL, BANCR, ENST00000537266, ENST00000426615, FAL1, H19, HIT000218960, LOC100507661, MALAT1, NONHSAT076747, NR_036575.1, and PVT1). In PTC, the deregulation of lncRNAs has been correlated with *BRAF* V600E mutation (BANCR, ENSG00000230498.1, ENSG00000273132.1, LOC100507661, NAMA, XLOC_006074, and XLOC_051122), determining clinical aggressiveness (ANRIL, ATB, BANCR, CASC2, CTD-3193013, ENSG00000415582, ENSG00000462717, FAL1, GAS5, H19, HIT000218960, HOTAIR, HOXD-AS1, LINC00271, LOC100507661, MALAT1, NONHSAT076747 and NONHSAT122730, NONHSAT037832, NONHSAT076754, NR_036575.1, PANDAR, PVT1, RP5-1024C24.1, RP11-40216.1, TCONS-00024700, XLOC_006074, and XLOC_051122), and RAI resistance (ENSG00000415582, ENSG00000462717, MEG3, NEAT1, and NR-028494) [96,97,98,99].

Similar to PTCs, though limited, there is published data documenting deregulation and the role of lncRNAs in FTC, PDTC, ATC, and MTC [96,97,98,99].

### 2.3. Familial Thyroid Cancer

Familial cancer is defined as one in which at least three first degree relatives are affected. It occurs due to an inherited mutation [100]. In the case of the thyroid, familial cancers usually occur at a younger age, and have been reported to be more aggressive than sporadic, with a higher risk of lymph node metastasis and extrathyroidal extension [101,102]. Familial thyroid tumors are more often encountered among MTC; therefore, they are broadly divided into familial non-MTC (follicular cell-derived) thyroid lesions and familial MTC (Table 1). 

Of the patients with a DTC, 5% have familial disease [101]. Familial follicular cell-derived tumors can occur as part of a syndrome primarily associated with non-thyroidal tumors, namely familial adenomatous polyposis (FAP), Gardner syndrome (a form of FAP), Cowden disease, Carney complex, Werner syndrome, Pendred syndrome, multiple endocrine neoplasia 1 (MEN 1), multiple endocrine neoplasia 2A (MEN 2A), Peutz–Jeghers syndrome, and DICER1 syndrome. Thyroid lesions that can develop in these familial cancer syndromes include nodular hyperplasia, FA, PTC (classic or follicular variant), FTC, and ATC. The cribriform-morular variant of PTC is typical of FAP and Gardner syndromes [101,102].

A second group of syndromes also exists, in which thyroid lesions are the primary manifestation [101]. These include familial follicular cell-derived thyroid carcinoma syndromes. These are not well characterized, but include familial PTC, a subset of which is associated with adenomatous nodules or papillary renal neoplasms, or shows prominent oxyphilia, or can be indistinguishable from classical PTC [101].

About 10–20% of MTC are familial [102] and the syndromes associated include Sipple’s syndrome (MEN 2 or 2A), MEN 2B, and familial MTC. Associated C cell hyperplasia on histopathology and *RET* gene mutations are characteristic [105].

### 2.4. Predisposition to Thyroid Cancer

Single nucleotide polymorphism (SNP) is, by definition, a variation that occurs within a single nucleotide of the DNA sequence, and which is identified in at least 1% of the population. Genome-wide association studies (GWAS) performed either at specific gene or at the whole genome level, identify SNPs associated with a certain disease. This has opened a new avenue for identifying genetic loci linked with cancers and other diseases. SNPs are, thus, markers of increased genetic susceptibility on a population level [106].

In thyroid, SNPs documented to be associated with DTC include rs1867277 (*FOXE1* or *TTF 2*), rs966423 (*DIRC3*), rs11693806 (*DIRC3*), rs12990503 (*DIRC3*), rs2439302 (*NRG1*), rs6996585 (*NRG1*), rs12542743 (*NRG1*), rs965513 (*FOXE1*), rs1867277 (*FOXE1*), rs72753537 (*FOXE1*), rs944289 (*NKX2-1*), rs34081947 (*NKX2-1),* rs116909374 (*MBIP1*), rs12129938 (*PCNXL2*), rs4649295 (*PCNXL2*), rs6793295 (*LRRC34*), s10069690 (*TERT*), rs73227498 (*EPB41L4A*), rs7902587 (*OBFC1*), rs2289261 (*SMAD3*), rs368187 (*LOC105370452*), rs1588635 (*PTCSC2*), and rs2466076 (*NRG1*) [107,108]; most of the data being derived from studies undertaken on European cohorts. While some of these SNPs have also been found to be associated with DTC in studies based on populations of a Korean, Chinese, and Japanese origin [109,110,111], other SNPs found in the Korean population include rs11175834 (*MSRB3*), rs4915076 (*VAV3*), rs1874564 (*SEPT11*), rs9858271 (*FHIT*), rs7248104 (*INSR*), and rs16934253 (*SLC24A6*) [107]. Interestingly, predisposing genetic factors documented by SNPs can be partly common for both benign and malignant follicular epithelium-derived thyroid tumors [112].

Genetic susceptibility has also been found for radiation-linked DTC. The SNPs implicated include rs965513 (*FOXE1*), rs71369530 (*FOXE1*), rs1867277 (*FOXE1*), rs3092993 (*ATM*), rs1801516 (*ATM*), rs2296675 (*MGMT*), rs2278420 (*ZNF350*), rs1991517 (*TSHR*), rs1799939 (*RET*), and rs1052559 (*ERCC2* or *XPD*) [113].

## 3. Preoperative Molecular Diagnosis of Indeterminate Thyroid Nodules

Ultrasound-guided FNA is a procedure of choice for evaluating thyroid nodules [2,114]. About 15% of all thyroid FNA samples are cytologically classified as indeterminate diagnostic categories, which include atypia of undetermined significance/follicular lesion of an undetermined significance and follicular neoplasm/suspicious for follicular neoplasm [115]. The 2015 American Thyroid Association guidelines recommend that molecular testing can reduce the number of repeat FNA and the rate of unnecessary diagnostic surgery in patients with indeterminate cytologic diagnosis [2,116].

**Figure 3:**
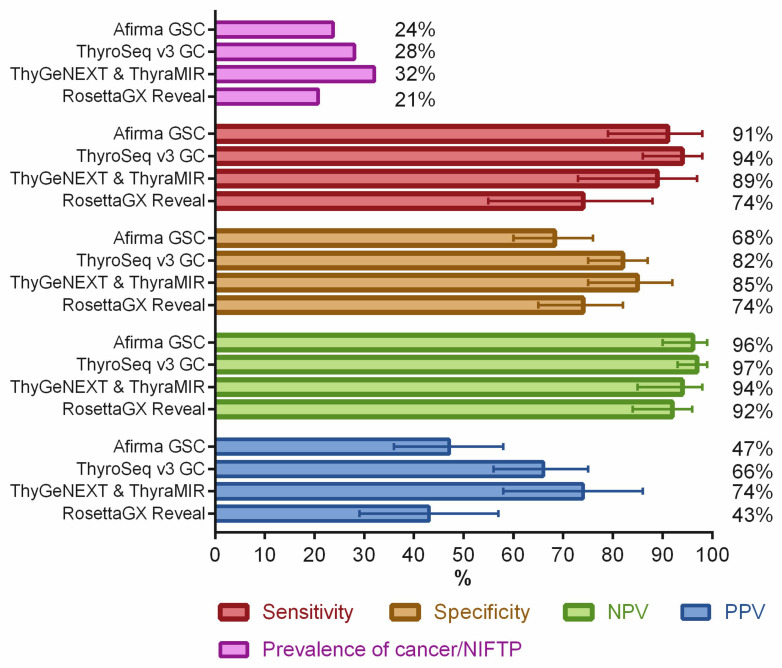
Diagnostic performance of commercially available molecular panels for thyroid nodules with indeterminate FNA cytology (atypia of undetermined significance/follicular lesion of undetermined significance and follicular neoplasm/suspicious for a follicular neoplasm). The length of the error bars is a 95% confidence interval. These data were obtained from clinical validation studies of Afirma Gene Sequencing Classifier (GSC) [118], ThyroSeq v3 Genomic Classifier (GC) [119], ThyGeNEXT and ThyraMIR [117], and RosettaGX Reveal [120]. NIFTP, non-invasive follicular thyroid neoplasm with papillary-like nuclear features; NPV, negative predictive value; and PPV, positive predictive value.

In thyroid nodules with indeterminate FNA cytology, the four main molecular tests commercially used in the United States are: ThyroSeq v3 Genomic Classifier (GC) (Sonic Healthcare, NY, USA); Afirma Gene Sequencing Classifier and Xpression Atlas (Veracyte, South San Francisco, CA, USA); ThyGeNEXT and ThyraMIR (Interpace Diagnostics, Parsippany, NJ, USA); and RosettaGX^®^ Reveal™ (Reveal) (Rosetta Genomics, Philadelphia, PA, USA). These molecular tests are characterized by NGS based the genotyping and gene expression profiling of mRNA or microRNA, and providing a high negative predictive value and risk stratification of cancer and NIFTP. These molecular tests were validated in prospectively collected samples [117,118,119] or in a retrospective blinded validation set [120], and their test performances are summarized in Figure 3.

## 4. Liquid Biopsy

Liquid biopsy is a non-invasive method used for early diagnosis, follow-up, and molecular profiling of cancer. A growing number of studies have evaluated the utility of liquid biopsy in thyroid cancer.

Circulating tumor cells have been found in thyroid cancers of both follicular and parafollicular cell origin. Limited evidence suggests that the number of circulating tumor cells correlates with initial tumor stage, and also acts as a predictor of recurrence, metastasis, and overall survival, although no definite cut-off values have been established. Attempts have also been made to test the utility of a decrease in circulating tumor cell count to predict response to RAI, but with inconclusive results [121].

Circulating cell-free DNA (cfDNA) or circulating tumor DNA (ctDNA) provides for an easily accessible source of tumor DNA for molecular evaluation. The utility of cfDNA or ctDNA for detecting alterations in a single gene, commonly *BRAF* V600E mutation, or multiple genes in thyroid cancer, has been evaluated. However, the sensitivity and specificity of the technique have varied across studies. Thus, liquid biopsy cannot yet replace tissue evaluation in endocrine tumors. It can, instead, be used for follow-up after the identification of specific mutations in the tissue [121]. Sato [122], in a recent study on 22 cases of PTC, concluded that *BRAF* V600E, when detected in pre-surgery plasma is indicative of a high fraction of *BRAF* V600E in the tumor, and extrathyroidal extension. In addition, the presence of the mutation in post-surgery ctDNA can be predictive of tumor recurrence [122]. As advanced disease and dedifferentiated thyroid cancer are more likely to have detectable ctDNA, its analysis is particularly useful in this group of patients for diagnosis, deciding upon targeted therapy, and follow-up [123,124]. A recent study on ATC also documented a worse overall survival, in patients with *PIK3CA* mutation detected in cfDNA [124].

In addition to circulating DNA, limited data exists on the use of circulating RNA as a potential source of detecting *BRAF* V600E mutation in blood [125].

Plasma-derived exosomes are alternative non-invasive biomarkers. Limited evidence suggests a change in the miRNA profile of exosomes with thyroid cancer development, and, hence, provides a potential tumor biomarker [126]. Plasma exosomes derived from cancer cells also act as a potential source of tumor miRNA, with implications similar to those with miRNA derived from tumor tissues [127]. Other potential biomarkers include circulating free lncRNA and miRNA, as already discussed.

## 5. Targeted Therapies in Thyroid Cancer

Several selective and multikinase inhibitors are currently approved, to treat advanced or treatment-refractory thyroid cancer (Table 2). Multikinase inhibitors act on two or more target molecules. Selective inhibitors act on a single target molecule that is hyperactive or mutant in cancer cells. Specific molecular targets currently available for the treatment of thyroid cancer include *BRAF*, *RET*, *MEK*, and *NRTK*.

## 6. Immunohistochemical Markers

Immunostaining is a long-used indispensable tool, for complementing routine techniques in elucidating differential diagnosis in surgical pathology. It provides for an easy, cheap, and widely available technique used for the identification of lineage or cell type in oncopathology.

Transcription factors TTF1 (NKX2.1), PAX8, and TTF2 (FOXE1) are involved in the development and functioning of the thyroid gland [129]. Their tissue-specificity makes them useful immunohistochemical markers for the identification of follicular cell differentiation. Thyroglobulin and sodium/iodide symporter are other such markers, and the former is one of the commonly used immunostains for the detection of thyroid tissue outside of the thyroid gland proper. Calcitonin and carcinoembryonic antigen serve as identifiers for C cell differentiation.

The recent development of novel markers, including mutation-specific markers and those with translational importance, has revolutionized the practice of IHC. These advancements have greatly affected thyroid clinical practice and research, too.

### 6.1. Ki-67

Ki-67 is the protein product of the gene *MKI67*, and is a commonly used immunohistochemical marker of proliferation. Its physiological function is the prevention of the merging of chromosomes into a single chromatin mass after the nuclear envelope has been disassembled [130]. The monoclonal antibody, MIB1, is the most widely used and validated clone for Ki-67 [131,132].

**Figure 4:**
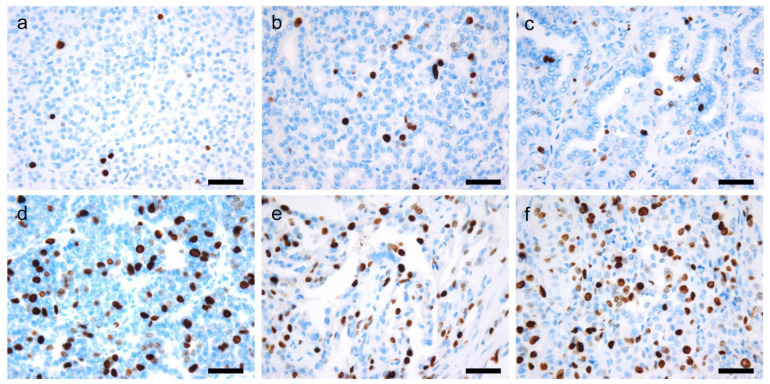
Immunohistochemical staining for Ki-67 in thyroid tumors. Different Ki-67 labeling indices are observed in follicular adenoma (**a**, 2%), follicular thyroid carcinoma (**b**, 4%), papillary thyroid carcinoma (**c**, 5%), poorly differentiated thyroid carcinoma (**d**, 20%), and high-grade papillary thyroid carcinoma (**e**, 35%) coexisting with anaplastic thyroid carcinoma (**f**, 40%). ×400 (**a**–**f**). Scale bar = 50 μm.

Compared to other organ systems, the Ki-67/MIB1 proliferative index is of limited use in thyroid pathology. This is because of the simplicity of diagnostic algorithms in contrast to other tumor classifications. Nevertheless, it does have utility in differentiating non-neoplastic from neoplastic thyroid lesions, and low-grade from high-grade tumors. Normal thyroid follicular cells show a Ki-67 index less than 0.1–0.2% [133,134]. In case of malignancies of follicular cell origin, the index increases with the decrease in differentiation: <10% in DTC, 10–30% in PDTC, and >30% in ATC [5] (Figure 4). A higher cut-off of (50%) has been recommended by some authors as a diagnostic criteria for ATC [135,136,137].

More recently, the Ki-67 index has been proposed to be useful in the stratification of PTC, FTC, and MTC into different risk categories, with a higher labeling index being associated with aggressive clinical behavior [135,138,139,140]. It has been proposed that DTC should be stratified into low-, moderate-, and high-risk groups using the Ki-67 cut-off values of <5%, 5–10%, and 10–30% [135,140].

In PTC, the cut-off values proposed have varied among studies, from 1 to 5% [135,141], with some authors suggesting that the Ki-67 index should be combined with other biomarkers to predict prognosis [141,142]. Importantly, a recent study demonstrated the Ki-67 index to correlate with the avidity of the tumor for RAI [143]. Aggressive histological variants of PTC are more likely to show a higher Ki-67 proliferative index than the classical variant, but have a better clinical outcome in the case of the presence of a low Ki-67 index [135]. Interestingly, the cribriform-morular variant of PTC, a relatively indolent tumor, has been reported to show a higher Ki-67 labeling index than classical PTC [144].

In minimally invasive FTC, there is limited evidence suggesting that a high Ki-67 index, defined as more than 5%, predicts tumor recurrence; although, an association with overall survival could not be demonstrated [138].

Similarly, in MTC, the Ki-67 index in combination with other biomarkers has been suggested to predict outcomes [139,145,146]. In a recent study, the authors defined a low Ki-67 proliferative index as <3%, intermediate as 3–20%, and high as >20% [139], while a two-tiered system considers a cut-off of 5% [147].

### 6.2. Second-Generation Neuroendocrine Markers

Chromogranin A and synaptophysin are the most widely used neuroendocrine markers. These, along with calcitonin and the carcinoembryonic antigen, are useful for the diagnosis of MTC. The second-generation neuroendocrine markers insulinoma-associated protein 1 (INSM1), ISL1, and secretagogin, have high sensitivity and specificity for neuroendocrine differentiation, expressed even in poorly differentiated neuroendocrine carcinomas [148,149,150]. INSM1 has been reported to be a highly sensitive and specific neuroendocrine marker, useful in the diagnosis of MTC and C cell hyperplasia [149].

Recently, FOXA1, a transcription factor involved in embryogenesis, has been found to be expressed in C cells and MTC. The absence of its expression in follicular cells makes it a useful marker for MTC diagnosis [151].

### 6.3. Next-Generation Immunohistochemistry

Next-generation IHC involves the use of antibodies to detect genetic alterations at the protein level [152], and is being increasingly applied in thyroid cancer, too (Table 3). Its main use is as a surrogate of molecular testing, which can have diagnostic, prognostic, and predictive significance.

#### 6.3.1. BRAF V600E (VE1)

**Figure 5:**
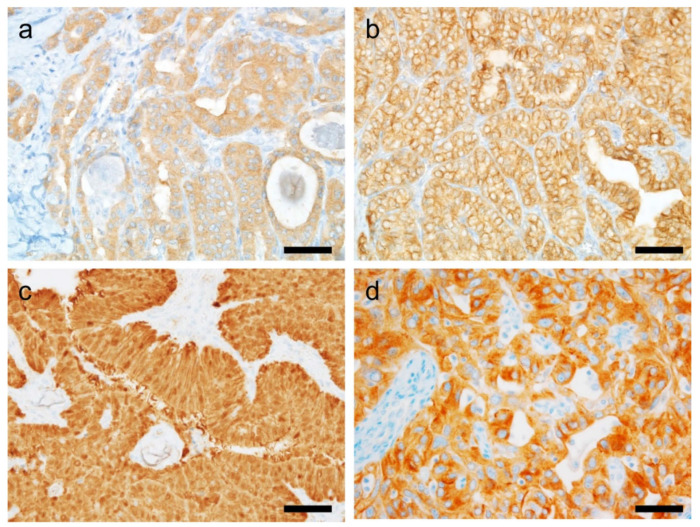
Immunohistochemical detection of mutations in thyroid cancer. (**a**) BRAF VE1 immunostaining in papillary thyroid carcinoma (PTC) with BRAF V600E mutation. (**b**) Pan-TRK immunostaining in PTC with RBPMS-NTRK3 fusion. (**c**) Cribriform morular thyroid carcinoma showing nuclear expression of β-catenin. (**d**) ALK immunostaining in PTC with EML4-ALK fusion. ×400 (**a**–**d**). Scale bar = 50 μm.

Immunostaining, using a mouse monoclonal antibody VE1, specific for the most prevalent mutation in thyroid cancer *BRAF* V600E, has shown excellent concordance with molecular testing (Figure 5a) [153,154,155]. A recent meta-analysis showed that by using this antibody, the pooled sensitivity and specificity of detecting *BRAF* V600E using VE1 is 96.8% and 86.3%, respectively [153]. The sensitivity and specificity of IHC depends on the sensitivity of the molecular test being used for confirmation, with maximum discordance (7–23%) being noted with Sanger sequencing, and the least (<2%) with real-time PCR [154]. Moreover, the fraction of discordant cases decreases to 3%, if two molecular tests are performed, and from 5% following a single molecular test [154]. However, before clinical use of the antibody in a given laboratory, the validation of the antibody using a reference molecular test in a well-powered pilot series is essential [155]. Appropriate positive control, such as a case of malignant melanoma proven by molecular testing to have *BRAF* V600E, should be used.

#### 6.3.2. RAS

Clone SP174 is a monoclonal *NRAS* Q61R mutation-specific antibody. It has also been documented to have high sensitivity and specificity for detecting other *RAS* mutations, namely *KRAS* *Q61R* and *HRAS* *Q61R* [156,157,158,159]. In MTC, *RAS* mutations occur as mutually exclusive from the germline *RET* mutation. Therefore, *RAS* mutation-specific IHC is useful for selecting patients with MTC for genetic testing [158]. The utility of SP174 immunostaining in the differential diagnosis of follicular-patterned thyroid neoplasms is, however, limited due to a lack of specificity of *RAS* mutations. These are present at variable frequencies in non-neoplastic and neoplastic thyroid lesions, including hyperplastic thyroid nodule, FA, FTC, NIFTP, and the follicular variant of PTC [11,157,160]. *RAS* mutated colorectal carcinoma or melanoma cases can be used as positive control.

#### 6.3.3. Pan-Trk

Immunostaining against TRK antigens detects increased levels of any of the three Trk proteins, namely, Trk A, Trk B, or Trk C (Figure 5b), and can be used as a screening tool, as it helps in cost-cutting; however, it is not acceptable as a stand-alone technique [26].

EPR17341 is the most widely used clone [161]. It detects the C-terminal region of all the three Trk proteins, and detects both wild-type and fusion proteins, and is, hence, considered pan-TRK [161]. IHC staining pattern is heterogeneous and, depending upon the specific fusion partner, can be nuclear, perinuclear, cytoplasmic granular, diffuse cytoplasmic, or membranous. There can be false negative and false positive results. The cut-off to categorize a case as positive, ranges from 1 to 10% [26]. Normal tissues of the central and peripheral nervous system, vascular smooth muscle, and ganglion cells in the wall of the appendix, serve as positive control [26].

#### 6.3.4. β-Catenin

The cribriform-morular variant of PTC can occur sporadically or more commonly in association with FAP. The tumor frequently harbors germline or somatic mutation of adenomatous polyposis coli (*APC*) or somatic mutation of *CTNNB1*, both of which lead to the accumulation of β-catenin in cytoplasm and nucleus of tumor cells, which can be detected by IHC (Figure 5c) [162]. Another PTC variant, PTC with fibromatosis/fasciitis-like stroma, demonstrates *CTNNB1* mutation in its mesenchymal component, detectable by IHC [163].

#### 6.3.5. PTEN

*PTEN* inactivation can be assessed immunohistochemically, with the loss of protein expression being indicative [164,165]. Barletta, in their study on 21 proven/suspected cases of Cowden syndrome, reported IHC to have a high sensitivity (100%) and specificity (92.3%) [166]. Beg et al. reported a lack of association between IHC results and *PTEN* gene deletion, as detected by FISH. The authors concluded that mechanisms, such as epigenetic modifications other than gene deletion, are involved in loss of PTEN protein expression [58].

#### 6.3.6. ALK

Increased ALK kinase activity, resulting from translocations and mutations involving the ALK gene, can be detected immunohistochemically [20,21] (Figure 5d). The four most commonly used and tested antibody clones against this protein include ALK1, 1A4, D5F3, and 5A4. Of these, D5F3 and 5A4 have been reported to have the highest sensitivity in detecting ALK rearrangements in lung cancer [167]. Ventana ALK D5F3 CDx assay (Ventana ALK (D5F3) CDx Assay, Ventana Medical Systems, Tucson, AZ, U.S.A.) has been approved by the FDA as a companion diagnostic and predictive kit for using crizotinib in patients with non-small cell lung cancer [168].

### 6.4. Tumor Microenvironment

Tumor development and progression are regulated by genetic and epigenetic changes in the tumor cells and the modulation of the tumor microenvironment (TME). Tumor cells dynamically interact with specific components of the TME and create favorable environments for immune escape, angiogenesis, tumor progression, and metastasis [169]. Immune cells in TME can exert both antitumor and protumor functions in thyroid cancer [170].

The immunosuppressive cells promoting tumor growth are regulatory T (Treg) cells, myeloid-derived suppressor cells (MDSCs), CD163+ (M2-type) tumor-associated macrophages (TAM), N2-type tumor-associated neutrophils (TAN), tumor-associated mast cells, and immature dendritic cells [171,172,173]. Treg infiltration in the TME occurs at a higher frequency in metastatic lymph nodes, locally advanced pT4 DTC, and ATC [171]. Increased TAM density in TME was associated with the lymph node metastasis of PTC [174], and reduced cancer-related survival in advanced thyroid cancer [175]. TAN density was associated with thyroid cancer size [176].

A higher ratio between peripheral blood neutrophil and lymphocyte count was associated with a larger tumor size and high risk of recurrence in DTC [177], and was more frequently found in ATC than in other types of thyroid cancers [173]. A high level of circulating MDSC in preoperative peripheral blood was associated with persistent disease after initial treatment [178].

Anticancer immune cells in thyroid cancers include cytotoxic CD8+ T cells (CTLs), natural killer (NK) cells, Th1 cells, M1 TAMs, N1 TANs, and mature dendritic cells [173]. A high CTL infiltration in tumor tissue was associated with improved disease-free survival of patients with DTC [179].

Cancer-associated fibroblasts (CAFs) as the major cellular components of the TME, play a key role in cancer development and progression [180]. The high expression of CAF markers, such as platelet-derived growth factor (PDGFR)-β, α-smooth muscle actin (α-SMA), and vimentin in tumor tissue, was associated with lymph node metastasis, *BRAF* V600E mutation, and shorter survival in PTC [180].

#### 6.4.1. PD-1/PD-L1

PD-1 (programmed cell death-1) is a receptor expressed on the surface of activated T cells. It acts as an immune checkpoint protein by interacting with PD-L1 (programmed cell death ligand-1) and PD-L2 present on the surface of other immune cells. This interplay regulates the T cell immune response in physiological conditions. PD-L1 is overexpressed in many cancers, and is being increasingly evaluated in thyroid cancer as a diagnostic, prognostic, and therapeutic marker.

**Figure 6:**
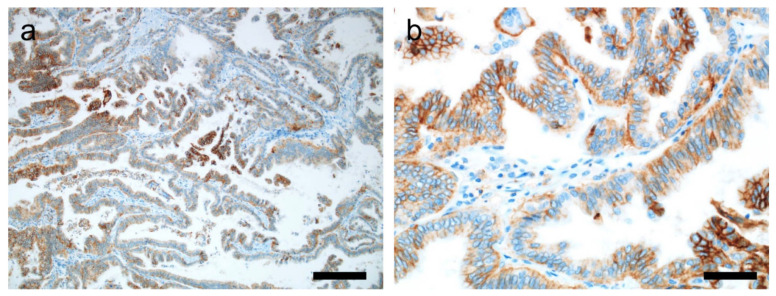
PD-L1 expression in papillary thyroid carcinoma. (**a**) Diffuse expression of PD-L1 on tumor cells (×100). (**b**) A high-power view shows the membranous staining for PD-L1 in cancer cells (×400). Scale bar = 50 μm.

PD-L1 expression determines the response to anti-PD-L1 therapy in various malignancies. Currently, immunotherapy as a part of combination therapy is being evaluated in metastatic and RAI-refractory thyroid cancer [181]. PD-L1 expression can be assessed at protein level by IHC (Figure 6), or at mRNA level [182].

Multiple PD-L1 clones are available for IHC [182]. The FDA has approved PD-L1 IHC using a rabbit monoclonal antibody (clone 22C3), for selecting patients for the PD-1-blocking drug pembrolizumab in metastatic non-small-cell lung cancer [183], Ventana PD-L1 (SP263) assay for treatment of patients with locally advanced or metastatic urothelial carcinoma with anti-PD-L1 immunotherapy durvalumab, and Ventana PD-L1 (SP142) IHC assay for detecting PD-L1 expression in tumor-infiltrating immune cells. Quite a few scoring systems have been proposed for the quantitation of PD-L1 expression in different malignancies [184]. The tumor proportion score (TPS) depicts the percentage of tumor cells showing partial or complete membranous positivity. The combined positivity score (CPS) is the proportion of positive tumor cells and intratumoral immune cells relative to the total number of tumor cells. Other scoring systems include the immune cell score, which takes into account only the tumor-infiltrating immune cells, and the tumor cell and immune cell area scores [185]. At present, there is no consensus regarding the preferred scoring system to be used in thyroid cancer.

The reported frequency of PD-L1 positivity in the tumor cells of different histological subtypes of thyroid cancer has ranged from 7% to 90% [186], and is variable even within the same histological type. This wide range is due to the differences in the clone (22C3, SP142, SP263, E1L3N, 4059, ab82059, ab174838, E1J2J, EPR1161-2, and 5H1); assay (manual vs automated and whole section vs tissue microarray); evaluation methods (membranous and/or cytoplasmic); the cut-off values used; and intratumoral heterogeneity. The optimal cut-off value for positivity of PD-L1 staining has not yet been validated in thyroid cancer, and values used have been more than 0%, 1%, 5%, 10%, 25%, or 30% [186,187]. It is essential that the reaction is evaluated for membranous expression and not for cytoplasmic positivity, as the former is a requisite for clinical trials, upon which anti-PD-1/PD-L1 therapy is also dependent.

Limited evidence suggests that PD-L1 expression can help to distinguish NIFTP from the invasive variant [182]; however, this finding has not been validated/replicated.

A recent meta-analysis documented PD-L1 expression to be significantly associated with autoimmune thyroiditis, *BRAF* V600E status, and reduced disease-free survival, but not with overall survival [186].

#### 6.4.2. CD Markers

The cluster of differentiation (also known as cluster of designation) antigens or CD markers are specific types of molecules expressed on the cell surface that help distinguish one cell type from another. These are most widely used in hematopathology to determine cell lineage.

**Figure 7:**
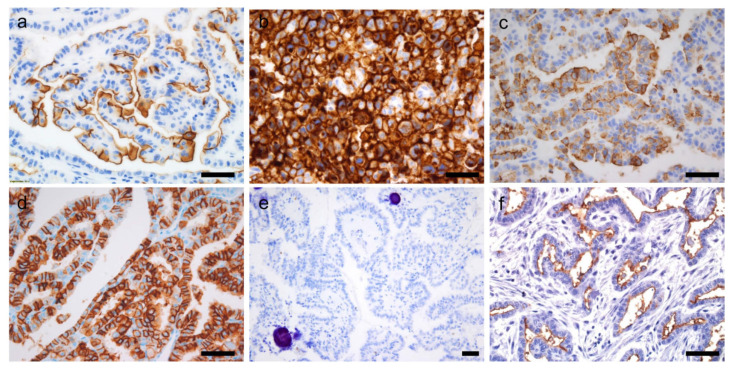
Immunohistochemical expression of CD markers by the thyroid cancer cells. CD10 expression in papillary thyroid carcinoma (PTC, **a**) and anaplastic thyroid carcinoma (**b**), CD15 expression in PTC (**c**), CD20 expression in PTC (**d**), loss of CD56 expression in PTC (**e**), and CD73 expression in PTC (**f**). ×400 (**a**–**d**,**f**). ×200 (**e**). Scale bar = 50 μm.

Normal thyroid tissue does not express CD10, CD15, CD20, CD57, CD73, CD99, and CD227. These CD markers are more frequently expressed in malignancy than in benign tumors (Table 4). CD5 is strongly expressed in intrathyroidal thymic carcinoma, but not in thyroid tumors derived from follicular or C cells. On the contrary, CD56 and CD117 are diffusely expressed in normal thyroid tissue and retained in benign thyroid diseases, but their expression is lost in thyroid cancers (Table 4; Figure 7). As with other immunohistochemical markers, there are the differences in the intensity and distribution of immunostaining within the tissues. An understanding of aberrant expression of CD markers in thyroid tumors is not only useful in the differential diagnosis of benign and malignant tumors, but it can also help pathologists to avoid diagnostic pitfalls in the diagnosis of metastatic tumors. It is important that CD markers can as serve as potential therapeutic targets.

CD47 (integrin associated protein) is a ubiquitously expressed “don’t eat me” marker. Its action is mediated by binding to the signal regulatory protein alpha (SIRPα), present on the surface of macrophages. When overexpressed in cancer cells, it inhibits tumor cell phagocytosis. Hence, targeting CD47 is being increasingly evaluated as a management option in ATC [188]. Roles in thyroid oncogenesis, PD-L1 signaling, and multidrug resistance have also been suggested [189,190].

### 6.5. Other IHC Markers with Potential Promise for Targeted Therapy

#### 6.5.1. PSMA

Prostate-specific membrane antigen (PSMA) is a marker of prostate epithelium, and is overexpressed in prostate cancer cells. Interestingly, it has been found to be expressed in endothelial cells of neovasculature in various malignancies and, hence, a possible role in theranostics [216,217].

In thyroid, PSMA expression in neovasculature has been reported to be more frequent in cancers, compared to benign tumors. Furthermore, tumor-associated vessels of PDTC and ATC show a higher expression of PSMA when compared with DTC [218,219]. In DTC, too, a strong PSMA expression is a predictor of shorter progression-free survival and refractoriness to RAI [220,221]. Hence, 68Ga-PSMA has translational relevance and can be used for the imaging and treatment of RAI-refractory thyroid carcinoma. However, potential pitfalls exist [218]. A variable low-level expression has been noted, even in non-neoplastic thyroid diseases within endothelial cells and in dendritic cells [218,219]. Notably, oncocytic tumors, including carcinomas, show low PSMA expression [218].

#### 6.5.2. MSI/MMR

Microsatellite instability (MSI), resulting from the inactivation of DNA mismatch repair (MMR), has been variably reported in thyroid carcinomas [222,223,224] and even in benign lesions, such as nodular goiter and FA [223]. Stepwise acquisition of mutations involving MMR genes with an increasing MSI, has been indicated as a possible pathway of thyroid tumor progression and dedifferentiation [223,225]. Interestingly, limited evidence suggests improved survival in ATC patients with MMR-deficient tumors than those with intact MMR profile [224].

IHC is a useful screening tool for evaluating MSI status, by using antibodies for detecting the MMR proteins MLH1, PMS2, MSH2, and MSH6. It has sensitivity and specificity similar to MSI [226], and has been evaluated in thyroid carcinomas, though to a limited extent [222].

## 7. Conclusions

High throughput NGS as a sensitive and accurate diagnostic tool, and liquid biopsy as a less invasive source of nucleic acid, have revolutionized the diagnosis and management of thyroid cancer, with the aim to achieve individualized treatment. There is an ever-growing list of novel and potential biomarkers, which are variably relevant for determining familial occurrence, establishing the diagnosis, targeted therapy, predicting clinical outcome, or even tumor response to therapy. Some of these include various genetic and epigenetic modifications, microRNAs, lncRNAs, germline mutations in MTC and nonMTC, and SNPs. Next-generation IHC is the best example of bench-to-bedside research. It provides for the assessment of genetic markers, namely BRAF V600E, RAS, TRK, PTEN, and ALK. It is more cost-effective, widely available, and easier to perform. Novel neuroendocrine and C cell markers, such as INSM-1, ISL1, secretagonin, and FOXA1, are upcoming in thyroid oncopathology practice and under investigation. While immunotherapy has developed in various cancers, its role in refractory thyroid cancer is still under investigation. An awareness of these advancements ensures their application in clinical practice, as well as facilitates research.

## Figures and Tables

**Table 1:** Familial thyroid tumors.

Familial Thyroid Cancer	Germline Mutation	Histology	References
Familial non-MTC	*HABP2*, *SRRM2*, *FOXE1*, *DUOX2*, *SRGAP1*, *TITF-1/NKX2.1*, *MNG1*, *PTCSC3*, and *CHEK2*	NH, FA, PTC, and FTC	[103,104]
Familial PTC with papillary renal cell neoplasia	*PRN*	PTC	[101,102,105]
Familial adenomatous polyposis	*APC*	PTC-CMV	[101,102,105]
Cowden syndrome	*PTEN*, *SDHB-D*, *PIK3CA*, *AKT1*, *KLLN*, and *SEC23B*	PTC-FV, FTC, FA, NH, and C-cell hyperplasia	[52,53,54,101,102,105]
Carney complex	*PRKAR1 α*	PTC, FTC, FA, and NH	[101,102,105]
Werner syndrome	*WRN*	PTC, FTC, and ATC	[101,102,105]
McCune–Albright syndrome	*GNAS*	PTC, FTC, and FA with papillary growth	[102]
DICER1 syndrome	*DICER1*	NH, PTC, and FTC	[39]
MEN and FMTC	*RET*	MTC	[102,105]

ATC, anaplastic thyroid carcinoma; FA, follicular adenoma; FTC, follicular thyroid carcinoma; MTC, medullary thyroid carcinoma; NH, nodular hyperplasia; PTC, papillary thyroid carcinoma; PTC-CMV, papillary thyroid carcinoma, cribriform morular variant; PTC-FV, papillary thyroid carcinoma, follicular variant; MEN, multiple endocrine neoplasia; and FMTC, familial medullary thyroid carcinoma.

**Table 2:** Targeted drugs approved by the Food and Drug Administration for thyroid cancer [128].

Drugs	Thyroid Cancers	Targets
Multikinase Inhibitors		
Sorafenib	RAI-refractory DTC	VEGFR, PDGFR, and BRAF
Lenvatinib	RAI-refractory DTC	VEGFR, FGFR, PDGFR, c-Kit, and RET
Vandetanib	MTC	VEGFR2, EGFR, and RET
Cabozantinib	MTC	c-MET, RET, VEGFR2, and AXL
BRAF kinase inhibitors		
Vemurafenib	*BRAF* V600E mutated cancer	BRAF V600E and CRAF-1
Dabrafenib	*BRAF* V600E mutated ATC	BRAF V600E and CRAF
MEK inhibitors		
Selumetinib	RAI-refractory DTC	MEK1 and MEK2
Trametinib combined with dabrafenib	ATC	MEK1 and MEK2
NTRK inhibitors		
Larotrectinib and entrectinib	*NTRK* fusion-positive cancer	TrkA, TrkB, and TrkC
RET kinase inhibitors		
Selpercatinib (LOXO-292)	*RET* mutation or fusion-positive cancer	RET, RET mutants V804M, and G810R
Pralsetinib (BLU-667)	Advanced or metastatic *RET*-mutant MTC and *RET*-fusion-positive thyroid cancer	RET, RET mutants V804L, V804M, M918T, and CCDC6-RET fusion

ATC, anaplastic thyroid carcinoma; DTC, differentiated thyroid carcinoma; MTC, medullary thyroid carcinoma; and RAI, radioactive iodine.

**Table 3:** Immunohistochemistry for the detection of molecular alterations in thyroid cancer.

Molecular Alteration	Target Protein (Clone)	Tumor Type	Utility
*BRAF* V600E	BRAF V600E (clone VE1)	Subset of PTC, PDTC, and ATC	Diagnostic, prognostic, and predictive
*APC* (germline or somatic) or *CTNNB1*	β-catenin	Cribriform-morular PTC and PTC with fibromatosis/ fasciitis-like stroma	Diagnostic
*RAS* mutations	Pan-RAS Q61R (clone SP174), including NRAS Q61R, KRAS Q61R, and HRAS Q61R	FA, OA, FTC, OCA, NIFTP, subset of PTC, hyperplastic nodules, and MTC	Diagnostic
*PTEN* inactivation	PTEN	PTEN hamartoma tumor syndrome, FA, FTC, follicular variant of PTC, NIFTP, hyperplastic nodules, PDTC, ATC, OA, and OCA	Diagnostic
*NTRK* rearrangements	Pan-TRK	PTC and secretory carcinoma	Diagnostic and predictive
*ALK* rearrangement	ALK (clones 5A4 and D5F3)	PTC, PDTC, ATC, and MTC	Diagnostic and predictive

ATC, anaplastic thyroid carcinoma; FA, follicular adenoma; OA, oncocytic adenoma; FTC, follicular thyroid carcinoma; OCA, oncocytic carcinoma; MTC, medullary thyroid carcinoma; NIFTP, non-invasive follicular thyroid neoplasm with papillary-like nuclear features; PDTC, poorly differentiated thyroid carcinoma; and PTC, papillary thyroid carcinoma.

**Table 4:** CD marker expression in thyroid lesions.

CD Marker	Gene Symbol	Gene Name	Alias Gene Symbols	Normal Thyroid	Benign Nodules	NIFTP	Malignancy	Prognostic Factor	References
CD5	*CD5*	CD5 molecule	*LEU1* and *T1*	0%	0%	0%	ITC (100%)	n/d	[191]
CD10	*MME*	Membrane metalloendopeptidase	*CALLA*, *CD10*, and *NEP*	0%	0–22%	n/d	PTC (30–47%, F), FTC (27%, F) ATC (96%, D), and MTC (0%)	n/s	[192,193,194]
CD15	*FUT4*	Fucosyltransferase 4	*CD15*, *FCT3A*, *ELFT*, and *FUC-TIV*	0%	0–10%	n/d	PTC (57–85%), FTC (4–40%), MTC (20%), and ATC (0%)	Excellent therapeutic outcomes to RAI in PTC	[192,195,196,197]
CD20	*MS4A1*	Membrane spanning 4-domains A1	*CD20*, *B1*, *Bp35*, and *MS4A2*	0%	0%	n/d	PTC (8–23%), PDTC (13%), ATC (0%), and MTC (0%)	n/s	[198,199]
CD30	*TNFRSF8*	TNF receptor superfamily member 8	*CD30*, *D1S166E*, and *KI-1*	0%	<40%	n/d	PTC (67%), FTC (7%), ATC (33%), and MTC (67%)	n/d	[200]
CD44	*CD44*	CD44 molecule (Indian blood group)	*MIC4*, *MDU2*, *MDU3*, *IN*, *MC56*, *Pgp1*, *CD44R*, *HCELL*, and *CSPG8*	0%	n/d	n/d	PTC (80%)	Shorter PFS in PTC	[201]
CD44v6				0%	30–40%	n/d	PTC (70–97%) FTC (80–90%), PDTC (55%), ATC (40–75%), and MTC (14%)	n/d	[202,203]
CD56	*NCAM1*	Neural cell adhesion molecule 1	*NCAM* and *CD56*	100%	>90%	10–100%	PTC (<20%) and FTC (20–90%)	n/s	[204,205,206]
CD57	*B3GAT1*	Beta-1,3-glucuronyltransferase 1	*CD57*, *LEU7*, *GlcAT-P*, *HNK-1*, and *NK-1*	0%	10–20%	85%	PTC (>90%), FTC (>90%)	n/d	[197,204,207]
CD73	*NT5E*	5′-nucleotidase ecto	*NT5*, *CD73*, *eN*, *eNT*, and *CALJA*	0%	n/d	n/d	PTC (72%)	Shorter RFS in PTC	[208]
CD99	*CD99*	CD99 molecule (Xg blood group)	*MIC2*	0%	0%	0%	SETTLE (75%)	n/d	[191,209]
CD117	*KIT*	KIT proto-oncogene and receptor tyrosine kinase	*PBT*, *CD117*, *SCFR*, and *C-Kit*	8–100%	8–100%	n/d	PTC (0–71%), FTC (47%), ATC (40%), ITC (100%), and SETTLE (75%)	n/s	[191,209,210,211,212]
CD166	*ALCAM*	Activated leukocyte cell adhesion molecule	*CD166* and *MEMD*	0%	n/d	n/d	PTC (12%)	Shorter PFS in PTC	[201]
CD227	*MUC1*	Mucin 1, cell surface associated	*PUM*, *MCKD1*, *CD227*, *PEM*, *ADMCKD*, *ADMCKD1*, *MCKD*, and *MCD*	6%	21–30%	n/d	PTC (49–80%), FTC (49%)	Adverse prognosis in PTC (conflicting data)	[213,214,215]

Gene names and symbols follow the guidelines of gene nomenclature by the Human Genome Organization (HUGO) Gene Nomenclature Committee (HGNC). D, diffuse staining; F, focal staining; RAI, radioactive iodine; RFS, recurrence-free survival; PFS, progression-free survival; n/d, no data; n/s, not significant; ITC, intrathyroid thymic carcinoma; and SETTLE, spindle epithelial tumor with thymus-like differentiation.

## Supplementary Materials

The following are available online at https://www.mdpi.com/article/10.3390/cancers14010204/s1, Table S1: detailed information about Figure 1.
