# Supplementary material for: Emerging Biomarkers in Thyroid Practice and Research
Source: Cancers (Basel). 2021 Dec 31;14(1):204. doi: 10.3390/cancers14010204 (PMC8744846; doi:10.3390/cancers14010204)
Supplement: Supplementary file 1 [file cancers-14-00204-s001.zip › cancers-1516564-supplementary.pdf]

*Supplementary Materials*

# Emerging Biomarkers in Thyroid Practice and Research

**Shipra Agarwal [1], Andrey Bychkov [2,*] and Chan-Kwon Jung [3,4,*]**

[1] Department of Pathology, All India Institute of Medical Sciences, New Delhi 110608, India; drshipra0902@gmail.com
[2] Kameda Medical Center, Department of Pathology, Chiba, Kamogawa 296-8602, Japan
[3] Department of Hospital Pathology, College of Medicine, The Catholic University of Korea, Seoul 06591, Korea
[4] Cancer Research Institute, College of Medicine, The Catholic University of Korea, Seoul 06591, Korea
* Correspondence: bychkov.andrey@kameda.jp (A.B.); ckjung@catholic.ac.kr (C.-K.J.)

**Table S1.** Detail information about Figure 1. Frequency and pattern of genetic alterations across thyroid tumors.

| Tumor Type | References |
|---|---|
| NH | [1–3] |
| FA functioning | [1,4] |
| FA | [1–10] |
| OA | [3,4,10–14] |
| HTT | [4,15,16] |
| NIFTP | [4,10,17,18] |
| FTC | [2,4-10,12,19–29] |
| OCA | [4,10–14,22,28,30–32] |
| PTC, East Asia | [7,10,20,23,26–28,33,34] |
| PTC, Western | [3,4,20,22,26–28,30,35-37] |
| PDTC | [4,9,22,23,26,30,38–40] |
| ATC | [4,9,20,22,23,26,28,38–42] |
| MTC | [22,26,43–46] |

NH, nodular hyperplasia; FA, follicular adenoma; OA, oncocytic adenoma; NIFTP, non-invasive follicular thyroid neoplasm with papillary-like nuclear features; HTT, hyalinizing trabecular tumor; FTC, follicular thyroid carcinoma; OCA, oncocytic carcinoma; PTC, papillary thyroid carcinoma; OCA, oncocytic carcinoma; PDTC, poorly differentiated thyroid carcinoma; ATC, anaplastic thyroid carcinoma.

## References

1. Ye, L.; Zhou, X.; Huang, F.; Wang, W.; Qi, Y.; Xu, H.; Yang, S.; Shen, L.; Fei, X.; Xie, J., et al. The genetic landscape of benign thyroid nodules revealed by whole exome and transcriptome sequencing. *Nat Commun* **2017**, *8*, 15533, doi:10.1038/ncomms15533.
2. Jeong, S.H.; Hong, H.S.; Lee, E.H.; Kwak, J.J.; Lee, J.Y. Analysis of RAS mutation in thyroid nodular hyperplasia and follicular neoplasm in a Korean population. *Endocrinol Diabetes Metab* **2018**, *1*, e00040, doi:10.1002/edm2.40.

3. Nikitski, A.V.; Nikiforova, M.N.; Yip, L.; Karslioglu-French, E.; Carty, S.E.; Nikiforov, Y.E. Can TP53-mutant follicular adenoma be a precursor of anaplastic thyroid carcinoma? *Endocr. Relat. Cancer* **2021**, *28*, 621-630, doi:10.1530/ERC-21-0095.
4. Acquaviva, G.; Visani, M.; Repaci, A.; Rhoden, K.J.; de Biase, D.; Pession, A.; Giovanni, T. Molecular pathology of thyroid tumours of follicular cells: a review of genetic alterations and their clinicopathological relevance. *Histopathology* **2018**, *72*, 6-31, doi:10.1111/his.13380.
5. Cameselle-Teijeiro, J.; Fachal, C.; Cabezas-Agricola, J.M.; Alfonsin-Barreiro, N.; Abdulkader, I.; Vega-Gliemmo, A.; Hermo, J.A. Thyroid Pathology Findings in Cowden Syndrome: A Clue for the Diagnosis of the PTEN Hamartoma Tumor Syndrome. *Am. J. Clin. Pathol.* **2015**, *144*, 322-328, doi:10.1309/AJCP84INGJUVTBME.
6. Jung, S.H.; Kim, M.S.; Jung, C.K.; Park, H.C.; Kim, S.Y.; Liu, J.; Bae, J.S.; Lee, S.H.; Kim, T.M.; Lee, S.H., et al. Mutational burdens and evolutionary ages of thyroid follicular adenoma are comparable to those of follicular carcinoma. *Oncotarget* **2016**, *7*, 69638-69648, doi:10.18632/oncotarget.11922.
7. Yoo, S.K.; Lee, S.; Kim, S.J.; Jee, H.G.; Kim, B.A.; Cho, H.; Song, Y.S.; Cho, S.W.; Won, J.K.; Shin, J.Y., et al. Comprehensive Analysis of the Transcriptional and Mutational Landscape of Follicular and Papillary Thyroid Cancers. *PLoS Genet.* **2016**, *12*, e1006239, doi:10.1371/journal.pgen.1006239.
8. Borowczyk, M.; Szczepanek-Parulska, E.; Debicki, S.; Budny, B.; Verburg, F.A.; Filipowicz, D.; Wieckowska, B.; Janicka-Jedynska, M.; Gil, L.; Ziemnicka, K., et al. Differences in Mutational Profile between Follicular Thyroid Carcinoma and Follicular Thyroid Adenoma Identified Using Next Generation Sequencing. *Int. J. Mol. Sci.* **2019**, *20*, 3126, doi:10.3390/ijms20133126.
9. Duan, H.; Liu, X.; Ren, X.; Zhang, H.; Wu, H.; Liang, Z. Mutation profiles of follicular thyroid tumors by targeted sequencing. *Diagn. Pathol.* **2019**, *14*, 39, doi:10.1186/s13000-019-0817-1.
10. Bae, J.S.; Jung, S.H.; Hirokawa, M.; Bychkov, A.; Miyauchi, A.; Lee, S.; Chung, Y.J.; Jung, C.K. High Prevalence of DICER1 Mutations and Low Frequency of Gene Fusions in Pediatric Follicular-Patterned Tumors of the Thyroid. *Endocr. Pathol.* **2021**, *32*, 336-346, doi:10.1007/s12022-021-09688-9.
11. Maximo, V.; Soares, P.; Lima, J.; Cameselle-Teijeiro, J.; Sobrinho-Simoes, M. Mitochondrial DNA somatic mutations (point mutations and large deletions) and mitochondrial DNA variants in human thyroid pathology: a study with emphasis on Hurthle cell tumors. *Am. J. Pathol.* **2002**, *160*, 1857-1865, doi:10.1016/S0002-9440(10)61132-7.
12. Corver, W.E.; Ruano, D.; Weijers, K.; Den Hartog, W.C.E.; Van Nieuwenhuizen, M.P.; De Miranda, N.; Van Eijk, R.; Middeldorp, A.; Jordanova, E.S.; Oosting, J., et al. Genome Haploidisation with Chromosome 7 Retention in Oncocytic Follicular Thyroid Carcinoma. **2012**, *7*, e38287, doi:10.1371/journal.pone.0038287.
13. Ganly, I.; Makarov, V.; Deraje, S.; Dong, Y.; Reznik, E.; Seshan, V.; Nanjangud, G.; Eng, S.; Bose, P.; Kuo, F., et al. Integrated Genomic Analysis of Hurthle Cell Cancer Reveals Oncogenic Drivers, Recurrent Mitochondrial Mutations, and Unique Chromosomal Landscapes. *Cancer Cell* **2018**, *34*, 256-270 e255, doi:10.1016/j.ccell.2018.07.002.
14. Gilani, S.M.; Ross, J.A.; Prasad, M.L.; Hammers, L.; Cai, G.; Adeniran, A.J. Molecular alterations in Hurthle cell neoplasms of thyroid: A fine needle aspiration cytology study with cytology-histology correlation. *Cancer Cytopathol.* **2020**, 10.1002/cncy.22370, doi:10.1002/cncy.22370.
15. Marchio, C.; Da Cruz Paula, A.; Gularte-Merida, R.; Basili, T.; Brandes, A.; da Silva, E.M.; Silveira, C.; Ferrando, L.; Metovic, J.; Maletta, F., et al. PAX8-GLIS3 gene fusion is a pathognomonic genetic alteration of hyalinizing trabecular tumors of the thyroid. *Mod. Pathol.* **2019**, *32*, 1734-1743, doi:10.1038/s41379-019-0313-x.
16. Nikiforova, M.N.; Nikiforov, Y.E.; Ohori, N.P. GLIS rearrangements in thyroid nodules: A key to preoperative diagnosis of hyalinizing trabecular tumor. *Cancer Cytopathol.* **2019**, *127*, 560-566, doi:10.1002/cncy.22163.
17. Pusztaszeri, M.; Bongiovanni, M. The impact of non-invasive follicular thyroid neoplasm with papillary-like nuclear features (NIFTP) on the diagnosis of thyroid nodules. *Gland Surg* **2019**, *8*, S86-S97, doi:10.21037/gs.2018.12.01.
18. Rossi, E.D.; Faquin, W.C.; Baloch, Z.; Fadda, G.; Thompson, L.; Larocca, L.M.; Pantanowitz, L. Noninvasive Follicular Thyroid Neoplasm with Papillary-Like Nuclear Features (NIFTP): Update and Diagnostic Considerations-a Review. *Endocr. Pathol.* **2019**, *30*, 155-162, doi:10.1007/s12022-019-9574-7.
19. Placzkowski, K.A.; Reddi, H.V.; Grebe, S.K.; Eberhardt, N.L.; McIver, B. The Role of the PAX8/PPARgamma Fusion Oncogene in Thyroid Cancer. *PPAR Res* **2008**, *2008*, 672829, doi:10.1155/2008/672829.
20. Pstrag, N.; Ziemnicka, K.; Bluyssen, H.; Wesoly, J. Thyroid cancers of follicular origin in a genomic light: in-depth overview of common and unique molecular marker candidates. *Mol. Cancer* **2018**, *17*, 116, doi:10.1186/s12943-018-0866-1.
21. Genutis, L.K.; Tomsic, J.; Bundschuh, R.A.; Brock, P.L.; Williams, M.D.; Roychowdhury, S.; Reeser, J.W.; Frankel, W.L.; Alsomali, M.; Routbort, M.J., et al. Microsatellite Instability Occurs in a Subset of Follicular Thyroid Cancers. *Thyroid* **2019**, *29*, 523-529, doi:10.1089/thy.2018.0655.
22. Panebianco, F.; Nikitski, A.V.; Nikiforova, M.N.; Nikiforov, Y.E. Spectrum of TERT promoter mutations and mechanisms of activation in thyroid cancer. *Cancer Med* **2019**, *8*, 5831-5839, doi:10.1002/cam4.2467.

23. Yoo, S.K.; Song, Y.S.; Lee, E.K.; Hwang, J.; Kim, H.H.; Jung, G.; Kim, Y.A.; Kim, S.J.; Cho, S.W.; Won, J.K., et al. Integrative analysis of genomic and transcriptomic characteristics associated with progression of aggressive thyroid cancer. *Nat Commun* **2019**, *10*, 2764, doi:10.1038/s41467-019-10680-5.
24. Legrand, M.A.; Raverot, G.; Nicolino, M.; Chapurlat, R. GNAS mutated thyroid carcinoma in a patient with Mc Cune Albright syndrome. *Bone Rep* **2020**, *13*, 100299, doi:10.1016/j.bonr.2020.100299.
25. Paulsson, J.O.; Wang, N.; Gao, J.; Stenman, A.; Zedenius, J.; Mu, N.; Lui, W.O.; Larsson, C.; Juhlin, C.C. GABPA-dependent down-regulation of DICER1 in follicular thyroid tumours. *Endocr. Relat. Cancer* **2020**, *27*, 295-308, doi:10.1530/ERC-19-0446.
26. Santoro, M.; Moccia, M.; Federico, G.; Carlomagno, F. RET Gene Fusions in Malignancies of the Thyroid and Other Tissues. *Genes (Basel)* **2020**, *11*, 424, doi:10.3390/genes11040424.
27. Yang, J.; Gong, Y.; Yan, S.; Chen, H.; Qin, S.; Gong, R. Association between TERT promoter mutations and clinical behaviors in differentiated thyroid carcinoma: a systematic review and meta-analysis. *Endocrine* **2020**, *67*, 44-57, doi:10.1007/s12020-019-02117-2.
28. Murugan, A.; Qasem, E.; Al-Hindi, H.; Alzahrani, A. Analysis of ALK, IDH1, IDH2 and MMP8 somatic mutations in differentiated thyroid cancers. *Molecular and Clinical Oncology* **2021**, *15*, doi:10.3892/mco.2021.2373.
29. Paulsson, J.O.; Rafati, N.; Dilorenzo, S.; Chen, Y.; Haglund, F.; Zedenius, J.; Juhlin, C.C. Whole-Genome Sequencing of Follicular Thyroid Carcinomas Reveal Recurrent Mutations in MicroRNA Processing Subunit DGCR8. *The Journal of Clinical Endocrinology & Metabolism* **2021**, 10.1210/clinem/dgab471, doi:10.1210/clinem/dgab471.
30. Ibrahimpasic, T.; Xu, B.; Landa, I.; Dogan, S.; Middha, S.; Seshan, V.; Deraje, S.; Carlson, D.L.; Migliacci, J.; Knauf, J.A., et al. Genomic Alterations in Fatal Forms of Non-Anaplastic Thyroid Cancer: Identification of MED12 and RBM10 as Novel Thyroid Cancer Genes Associated with Tumor Virulence. *Clin. Cancer Res.* **2017**, *23*, 5970-5980, doi:10.1158/1078-0432.ccr-17-1183.
31. Gopal, R.K.; Kübler, K.; Calvo, S.E.; Polak, P.; Livitz, D.; Rosebrock, D.; Sadow, P.M.; Campbell, B.; Donovan, S.E.; Amin, S., et al. Widespread Chromosomal Losses and Mitochondrial DNA Alterations as Genetic Drivers in Hürthle Cell Carcinoma. *Cancer Cell* **2018**, *34*, 242-255.e245, doi:10.1016/j.ccell.2018.06.013.
32. Corver, W.E.; Morreau, H. Unique landscape of widespread chromosomal losses in Hurthle cell carcinoma. *Endocr. Relat. Cancer* **2019**, *26*, L1-L3, doi:10.1530/ERC-18-0481.
33. Cho, U.; Oh, W.J.; Bae, J.S.; Lee, S.; Lee, Y.S.; Park, G.S.; Lee, Y.S.; Jung, C.K. Clinicopathological features of rare BRAF mutations in Korean thyroid cancer patients. *J. Korean Med. Sci.* **2014**, *29*, 1054-1060, doi:10.3346/jkms.2014.29.8.1054.
34. Kim, S.Y.; Kim, T.; Kim, K.; Bae, J.S.; Kim, J.S.; Jung, C.K. Highly prevalent BRAF V600E and low-frequency TERT promoter mutations underlie papillary thyroid carcinoma in Koreans. *Journal of Pathology and Translational Medicine* **2020**, *54*, 310-317, doi:10.4132/jptm.2020.05.12.
35. Cancer Genome Atlas Research, N. Integrated genomic characterization of papillary thyroid carcinoma. *Cell* **2014**, *159*, 676-690, doi:10.1016/j.cell.2014.09.050.
36. Seethala, R.R.; Chiosea, S.I.; Liu, C.Z.; Nikiforova, M.; Nikiforov, Y.E. Clinical and Morphologic Features of ETV6-NTRK3 Translocated Papillary Thyroid Carcinoma in an Adult Population Without Radiation Exposure. *Am. J. Surg. Pathol.* **2017**, *41*, 446-457, doi:10.1097/PAS.0000000000000814.
37. Chou, A.; Fraser, S.; Toon, C.W.; Clarkson, A.; Sioson, L.; Farzin, M.; Cussigh, C.; Aniss, A.; O'Neill, C.; Watson, N., et al. A detailed clinicopathologic study of ALK-translocated papillary thyroid carcinoma. *Am. J. Surg. Pathol.* **2015**, *39*, 652-659, doi:10.1097/PAS.0000000000000368.
38. Ricarte-Filho, J.C.; Ryder, M.; Chitale, D.A.; Rivera, M.; Heguy, A.; Ladanyi, M.; Janakiraman, M.; Solit, D.; Knauf, J.A.; Tuttle, R.M., et al. Mutational profile of advanced primary and metastatic radioactive iodine-refractory thyroid cancers reveals distinct pathogenetic roles for BRAF, PIK3CA, and AKT1. *Cancer Res.* **2009**, *69*, 4885-4893, doi:10.1158/0008-5472.CAN-09-0727.
39. Landa, I.; Ibrahimpasic, T.; Boucai, L.; Sinha, R.; Knauf, J.A.; Shah, R.H.; Dogan, S.; Ricarte-Filho, J.C.; Krishnamoorthy, G.P.; Xu, B., et al. Genomic and transcriptomic hallmarks of poorly differentiated and anaplastic thyroid cancers. *J. Clin. Invest.* **2016**, *126*, 1052-1066, doi:10.1172/JCI85271.
40. Prete, A.; Matrone, A.; Gambale, C.; Torregrossa, L.; Minaldi, E.; Romei, C.; Ciampi, R.; Molinaro, E.; Elisei, R. Poorly Differentiated and Anaplastic Thyroid Cancer: Insights into Genomics, Microenvironment and New Drugs. *Cancers (Basel)* **2021**, *13*, 3200, doi:10.3390/cancers13133200.
41. Ljubas, J.; Ovesen, T.; Rusan, M. A Systematic Review of Phase II Targeted Therapy Clinical Trials in Anaplastic Thyroid Cancer. *Cancers (Basel)* **2019**, *11*, 943, doi:10.3390/cancers11070943.
42. Dias-Santagata, D.; Lennerz, J.K.; Sadow, P.M.; Frazier, R.P.; Govinda Raju, S.; Henry, D.; Chung, T.; Kherani, J.; Rothenberg, S.M.; Wirth, L.J. Response to RET-Specific Therapy in RET Fusion-Positive Anaplastic Thyroid Carcinoma. *Thyroid* **2020**, *30*, 1384-1389, doi:10.1089/thy.2019.0477.
43. Kim, H.J.; Jee, K.J.; Shong, Y.K.; Hong, S.J.; Gong, G. DNA copy number changes in thyroid medullary carcinomas determined by comparative genomic hybridization. *Korean J. Pathol.* **2008**, *42*, 27-32.

44. Grubbs, E.G.; Ng, P.K.; Bui, J.; Busaidy, N.L.; Chen, K.; Lee, J.E.; Lu, X.; Lu, H.; Meric-Bernstam, F.; Mills, G.B., et al. RET fusion as a novel driver of medullary thyroid carcinoma. *J. Clin. Endocrinol. Metab.* **2015**, *100*, 788-793, doi:10.1210/jc.2014-4153.
45. Ciampi, R.; Romei, C.; Ramone, T.; Prete, A.; Tacito, A.; Cappagli, V.; Bottici, V.; Viola, D.; Torregrossa, L.; Ugolini, C., et al. Genetic Landscape of Somatic Mutations in a Large Cohort of Sporadic Medullary Thyroid Carcinomas Studied by Next-Generation Targeted Sequencing. *iScience* **2019**, *20*, 324-336, doi:10.1016/j.isci.2019.09.030.
46. Maciel, R.M.B.; Maia, A.L. GLOBAL ENDOCRINOLOGY: Geographical variation in the profile of RET variants in patients with medullary thyroid cancer: a comprehensive review. *European Journal of Endocrinology* **2022**, *186*, R17-R32, doi:10.1530/eje-21-0753.
